# Navigating the Collagen Jungle: The Biomedical Potential of Fiber Organization in Cancer

**DOI:** 10.3390/bioengineering8020017

**Published:** 2021-01-21

**Authors:** Jonathan N. Ouellette, Cole R. Drifka, Kelli B. Pointer, Yuming Liu, Tyler J Lieberthal, W John Kao, John S. Kuo, Agnes G. Loeffler, Kevin W. Eliceiri

**Affiliations:** 1 Department of Biomedical Engineering, University of Wisconsin-Madison, Madison, WI 53706, USA; jouellette@wisc.edu (J.N.O.); crdrifka@gmail.com (C.R.D.); tyler.lieberthal@gmail.com (T.J.L.); wjkao@hku.hk (W.J.K.); 2 Laboratory for Optical and Computational Instrumentation, Center for Quantitative Cell Imaging, University of Wisconsin-Madison, Madison, WI 53706, USA; kelli.pointer@gmail.com (K.B.P.); liu372@wisc.edu (Y.L.); 3 Department of Industrial and Manufacturing Systems Engineering, Faculty of Engineering, University of Hong Kong, Pokfulam, Hong Kong; 4 Department of Neurosurgery, Dell Medical School, The University of Texas at Austin, Austin, TX 78712, USA; john.kuo@austin.utexas.edu; 5 Department of Pathology, MetroHealth Medical Center, Cleveland, OH 44109, USA; aloeffler@metrohealth.org; 6 Department of Medical Physics, University of Wisconsin-Madison, Madison, WI 53705, USA; 7 Morgridge Institute for Research, Madison, WI 53715, USA

**Keywords:** fibrillar collagen, cancer, prognosis, tumor microenvironment, extracellular matrix, ECM, pathology

## Abstract

Recent research has highlighted the importance of key tumor microenvironment features, notably the collagen-rich extracellular matrix (ECM) in characterizing tumor invasion and progression. This led to great interest from both basic researchers and clinicians, including pathologists, to include collagen fiber evaluation as part of the investigation of cancer development and progression. Fibrillar collagen is the most abundant in the normal extracellular matrix, and was revealed to be upregulated in many cancers. Recent studies suggested an emerging theme across multiple cancer types in which specific collagen fiber organization patterns differ between benign and malignant tissue and also appear to be associated with disease stage, prognosis, treatment response, and other clinical features. There is great potential for developing image-based collagen fiber biomarkers for clinical applications, but its adoption in standard clinical practice is dependent on further translational and clinical evaluations. Here, we offer a comprehensive review of the current literature of fibrillar collagen structure and organization as a candidate cancer biomarker, and new perspectives on the challenges and next steps for researchers and clinicians seeking to exploit this information in biomedical research and clinical workflows.

## 1. Introduction

**Figure 1:**
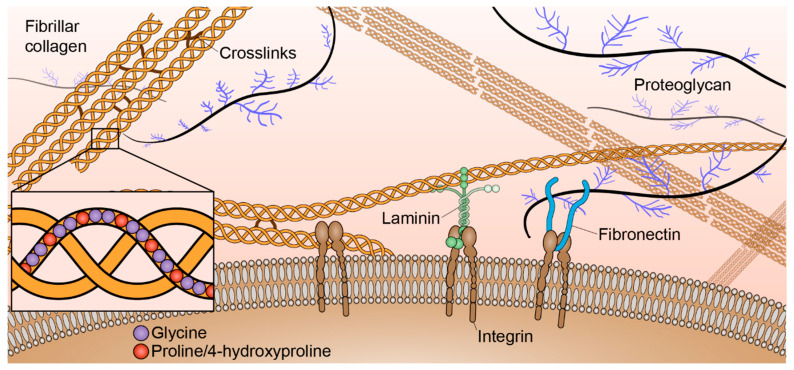
Key extracellular matrix (ECM) components of the tumor microenvironment. Collagens, laminin, fibronectin, proteoglycans, and other components are produced by fibroblasts and other resident cells.

The tumor microenvironment consists of multiple biochemical, mechanical, and structural signals. One of the major structural components of the tumor microenvironment is the extracellular matrix (ECM). The ECM is a very dynamic structure consisting of many components including collagen, laminin, fibronectin, glycoproteins, proteoglycans, and polysaccharides (Figure 1). There have been increasing efforts to better understand the influences of the ECM components on cell behaviors and functions. A major focus of studying the ECM is the role of collagen in both normal and abnormal function. Collagen is the most abundant ECM protein in the human body. There are twenty-eight different types of collagen involved in many normal biological functions such as tissue scaffolding, cell adhesion, cell migration, angiogenesis, tissue morphogenesis, and tissue repair [1]. Based on function and domain homology [2], these collagens are classified into seven groups: fibril-forming (fibrillar) collagens, fibril-associated collagens with interrupted triple helices, network-forming collagens, transmembrane collagens, endostatin-producing collagens, anchoring fibrils, and bead-filament-forming collagens.

Fibril-forming collagens, in particular, are highly abundant throughout the stroma. Normally, fibrillar collagens maintain tissue integrity and are separated from epithelial cells by a thin basement membrane (Figure 1). There are eleven fibrillar collagen genes that break down into seven different collagen types: type I, II, III, V, XI, XXIV, and XXVII. Molecular assembly of fibrillar collagen is hierarchical [3]. All collagen molecules consist of three polypeptide alpha chains (~1.6 nm width, ~300 nm length) that make at least one triple helical domain. The combination of three alpha chains determines the collagen type. These alpha chains consist of around 1,000 amino acid residues with a characteristic triplet repeat sequence Gly-X-Y, alpha chain types are differentiated by the amino acid residues in the X and Y positions of those triplets [4]. The most abundant fibrillar collagen, type I, is generally heterotrimeric and composed of two α1(I) and one α2(I) chains; however, a homotrimeric α1(I) isoform has been found to be produced by certain malignant cells and is resistant to proteolysis [5]. Collagen types II, III, XXIV, and XXVII are composed of homotrimers. Collagen types V and XI are often found as heterotrimeric molecules; however, homotrimers of the α1(V) chain have been characterized and composite molecules of type V and XI collagens have also been described. α3(XI) chains also appear to be a modified product of the gene that encodes the type II collagen chain [2]. Procollagen containing N- and C-propeptides at each end of the triple helical domains are ultimately processed into collagen upon protease cleavage [6]. Collagen molecules associate both in lateral and longitudinal directions to form collagen fibrils (~100 nm width, ~1 µm length) that are stabilized by non-reducible covalent cross-links that involve residues in the triple helix. Multiple fibrils can then aggregate to form collagen fibers (~1 µm width, ~10 µm length).

Collagen fiber formation is essential to human health. Abnormalities in collagen expression or collagen structure can result in debilitating disease. Osteogenesis imperfecta, for example, is a connective tissue disorder characterized by bone deformities, brittle bones, and low bone density caused by the improper encoding and assembly of collagen type I [7]. Over the last decade, there has been growing evidence that collagen fiber organization is not only a structural scaffold for normal tissue function, but, in some instances, it correlates with disease onset and progression. This review presents the current understanding of the role of collagen in oncogenesis.

**Figure 2:**
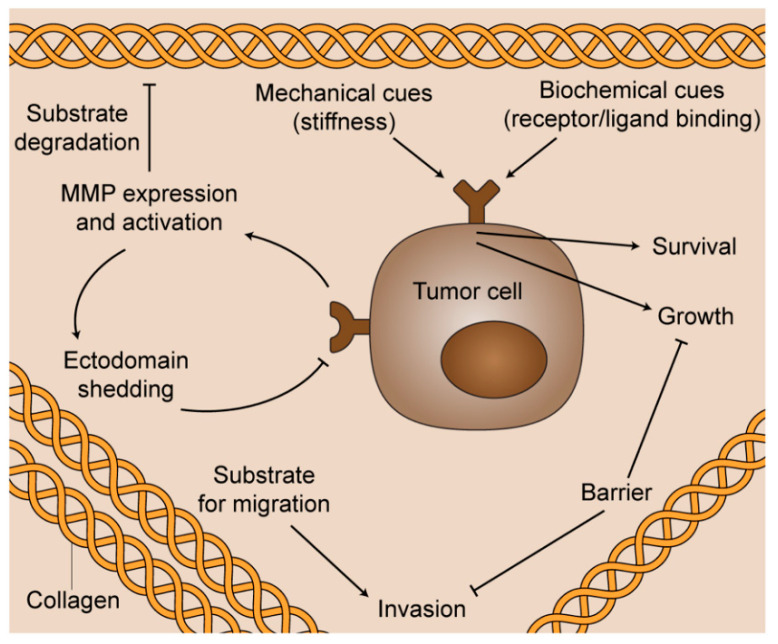
The multifunctional role of fibrillar collagen in cancer progression. Biochemical cues and specific organizational changes in collagen result in diverse pathological consequences for the survival, proliferation, and spread of cancerous cells.

During cancer progression, homeostatic control of the ECM is compromised, and epithelial cells become exposed to a collagen-rich stroma. In addition to elevated content, stromal collagen is differentially organized in a number of cancer types [8,9,10]. Both biochemical cues and specific organizational changes in collagen result in many pathological consequences (Figure 2). For example, breast cancer cells migrate in vivo along linearized collagen fibers aligned perpendicular to the stroma-cancer interface [8,11]. Subsequent mechanistic work demonstrated that aligned collagen limits cellular protrusions, and therefore increases migratory persistence [12]. Aligned collagen prevents T cells from migrating to tumor islets in human lung cancer [13]. Additionally, strain-stiffening behavior of collagen along the longitudinal axis contributes significantly to the tensile strength of tissue, and cancer cells respond aberrantly to aligned, stiff matrices via integrin signaling [14,15,16,17,18,19,20,21]. Aligned collagen also significantly contributes to increased stromal density and intratumoral fluid pressures [22], which can impede the transport of therapeutic agents to the tumor target site [23,24]. While the exact mechanisms remain incompletely understood, collagen alignment is believed to be produced and maintained by both cancer cells and stromal components through a number of biological underpinnings, including Rho-dependent actin-myosin contractility [11,25], intra- and intermolecular cross-linking via lysyl oxidase [17], syndecan-1 expression [26], and interactions among other ECM molecules such as fibronectin, periostin, and minor collagens [27,28,29,30,31,32].

In this review, we focus on the published evidence showing that fibrillar collagen organization and structure is an important factor and potential candidate biomarker in disease etiology and progression in a wide variety of cancers. We review the literature pertaining to collagen morphology in diagnosis, patient prognosis, and treatment response in many cancer types. We have primarily constrained this review to studies of human cancer tissue where fibrillar collagen structure and organization are visualized and measured using standard histopathologic specimen preparation methods (tissues from paraffin-embedded blocks of tissue).

## 2. Collagen Fiber Imaging and Quantification

While ongoing research seeks to fully understand the mechanistic causes and pathological consequences of collagen organization in cancer, much of the foundation of this work was established through imaging studies of pathological tissues from human patients. The role of fibrillar collagen organization in cancer is increasingly better understood because of the various imaging techniques and corresponding quantification tools that allow for examination of archival tissue cancer samples. We briefly review below some of the imaging and computational approaches that meet the spatial resolution and other quantification requirements for fibrillar collagen evaluation in cancer.

### 2.1. Histological Staining

Traditional histological staining methods such as Masson’s Trichrome, Movat’s Pentachrome, and Van Gieson are widely used to visualize collagen in thin sections (typically 5 micron) due to their low cost and relative ease of application. Picrosirius red is another staining technique used to detect collagen that, when combined with polarized light, is sensitive to fibrillar collagen birefringence [33] and, in the quantification of certain types of collagen fiber metrics, it was correlated well with Second Harmonic Generation (SHG) imaging which is a gold standard fibrillar collagen imaging technique and will be described below [34]. Picrosirius Red provides better specificity for collagen detection compared to the other collagen stains as differences in interference color and birefringent intensity can help further identify collagen subtypes [35]. Another new stain named collagen hybridizing peptide (CHP) was found to be effective for detection of degraded collagen in tissue samples and has the ability to target some types of collagen remodeling [36,37]. All of these stains, however, are limited in their clinical utility due to the variable quality of the stains, even with the use of automated platforms for staining. Antibody labeling has also been used extensively for collagen detection and characterization [38], but is complicated by cross-reactivity between collagen types. Despite the recognition that visible changes in the ECM (so-called “desmoplastic stroma”) are required for a diagnosis of invasive cancer in most epithelial malignancies, the possibility that the ECM holds any further clues to oncologic growth, prognosis, or treatment has not been explored by traditional staining methods. This is partly due to the fact that in standard clinical practice, collagen histological stains enable enhanced qualitative assessment of tissue specimens but are not readily amenable to collagen quantification.

### 2.2. Second Harmonic Generation Microscopy

There continues to be great interest in label-free imaging methods for tumor tissues that do not require specialized staining. SHG microscopy is a powerful optical method to non-invasively detect fibrillar collagen changes in intact tissues that cannot be readily achieved using biochemical or other imaging approaches [39,40,41]. SHG is a laser scanning microscopy technique in which two lower energy photons are up-converted to exactly twice the incident frequency [42]. For this phenomenon to occur, a non-centrosymmetric molecular structure is required. Fibrillar collagen and other biological molecules such as microtubules and muscle myosin satisfy this structural requirement [43]. SHG has a distinct advantage over traditional histological staining-based approaches because there is no need to process or stain the tissue, and the ability to optically section tissue allows for thicker specimens (several hundred microns or more) to be used. In addition, SHG is a high-resolution imaging technique allowing for submicron resolution, with down to ~300 nm views of collagen structures. SHG microscopy has emerged as the experimental “gold standard” for fibrillar collagen studies enabling collagen organization to be directly visualized and quantified in live [44] and fixed histology tissues [10,45,46], three-dimensional (3D) in vitro systems [12,47,48,49,50,51], and dynamic in vivo disease models [52,53]. However, the specialized equipment, significant cost, requisite specialized training. and computational requirements do not currently permit easy adoption of this methodology in routine clinical pathologic, radiologic, or surgical practices.

### 2.3. Liquid Crystal-Based Polarizing Microscope (LC-PolScope)

Polarization imaging methods, such as the liquid crystal-based polarization microscopy method LC-PolScope, is another label-free imaging modality for collagen. LC-PolScope is a simpler, less expensive, faster and hence a more clinically friendly imaging modality compared to SHG [54,55]. However, LC-PolScope imaging can only be used to assess collagen orientation in thin and transparent specimens. It has been shown to yield comparable orientation and alignment results from computed retardance images for traditional 5 micron histopathology sections including human breast cancer and pancreatic cancer histology slides [56]. Regular research-grade or clinical microscopes can readily be converted to a basic LC-PolScope by adding a universal compensator and a circular polarizer or analyzer. The universal compensator is made of two liquid crystal variable retarders and a linear polarizer, which can produce any light polarization. The LC-PolScope allows for fast measurements of specimen anisotropy (i.e., retardance and slow axis orientation) at all points of the image constituting the field of view. Nevertheless, an ongoing challenge of using LC-PolScope for collagen imaging is how to properly interpret the signals as they may represent other biological structures of birefringent contrast such as smooth muscle. While LC-Polscope is promising for collagen fiber assessment, the meaning inherent in the images produced by the LC-PolScope must first be validated in well-informed and well-executed studies.

### 2.4. Computational Methods

In addition to imaging the collagen topology, whether by traditional histological staining or by advanced imaging methods, there is the common need to quantitate collagen organization. A number of groups, including our own, have investigated the best computational approaches to quantify changes in collagen fiber organization in a wide range of biomedical applications. The main features that were found to be meaningful to date are collagen fiber amount or density [57,58], orientation and anisotropy of orientations (or alignment) [46,59], individual fiber properties including angle, width, length and curvature, texture analysis-based collagen fiber patterns [10,60,61,62], fiber network branching [63], and features related to combined analysis of collagen fibers and their associated tumor cells such as tumor-associated collagen signatures [8,46,64]. Among all of these features, collagen fiber orientation and alignment were of most interest to investigators. Pixel-wise orientation and window-wise orientation can efficiently be computed based on intensity derivatives or intensity variations [65,66] and transformation-based analysis [67,68,69,70,71,72] such as Fourier transform and Hough transform, respectively. Besides describing orientation-related features, approaches based on fiber-wise information [73,74,75] can provide data on many other morphologic features, but they are generally computationally demanding. To be noted, curvelet transform [76] can yield an optimal multiscale directional representation of the collagen fiber image, and has been used in our quantification studies to directly track local fiber orientation change or enhance fiber edges for later individual fiber extraction [77]. Machine learning has emerged as a powerful tool to identify discriminative fiber features [78] and can classify images into pre-determined categories (e.g., normal or abnormal tissues, lower and higher grades of cancer) based on either explicitly calculated fiber features [79] or implicit fiber patterns [80,81,82] in the collagen fiber images. Automated quantification approaches are promising to improve assessment accuracy of prognostic variables in clinical pathologic practice, and also expand research possibilities by enabling the measurement of larger areas of interest and greater numbers of samples than with current, manually intensive imaging technologies. Some most frequently cited or recently emerged open-source tools include Fiji plugins of OrientationJ [66], Ridge Detection [83], FibrilTool [84], TWOMBLI [85], MATLAB-based CytoSpectre [68], CurveAlign [77,86,87] and CT-FIRE [75,77], and Python-based PyFibre [79]. Users are recommended to follow the tutorials or protocols to test and choose a tool that best meets their needs. The validation of the usage or the accuracy of a tool usually relies on visual inspection, cross-validation by other computational tools developed based on different methodologies, or by comparing the results with those from manual or semi-automatic measurements. As well it can be useful to analyze computationally generated fiber images with known fiber metrics as shown in our previous publication [77]. Visual inspection, if available, is often the most practical strategy.

## 3. Collagen Fiber Organization as a Candidate Cancer Biomarker

Pathology-driven studies of human patient tissues have spotlighted collagen fiber organization as an intrinsic biomarker in oncologic diseases with potential clinical applications. Recent studies have demonstrated that discrete features of collagen fibers including width [88,89,90,91], length [10,90,92], angle [8], and alignment [59,86,93,94,95,96,97], can be used to differentiate benign from malignant tissues or serve as accurate predictors of cancer aggression and patient survival, while total tissue collagen content may be less important in determining cancer extent [98]. Insights from these studies are highlighted in Table 1 and reviewed below.

### 3.1. Tissue Diagnosis and Grading

**Figure 3:**
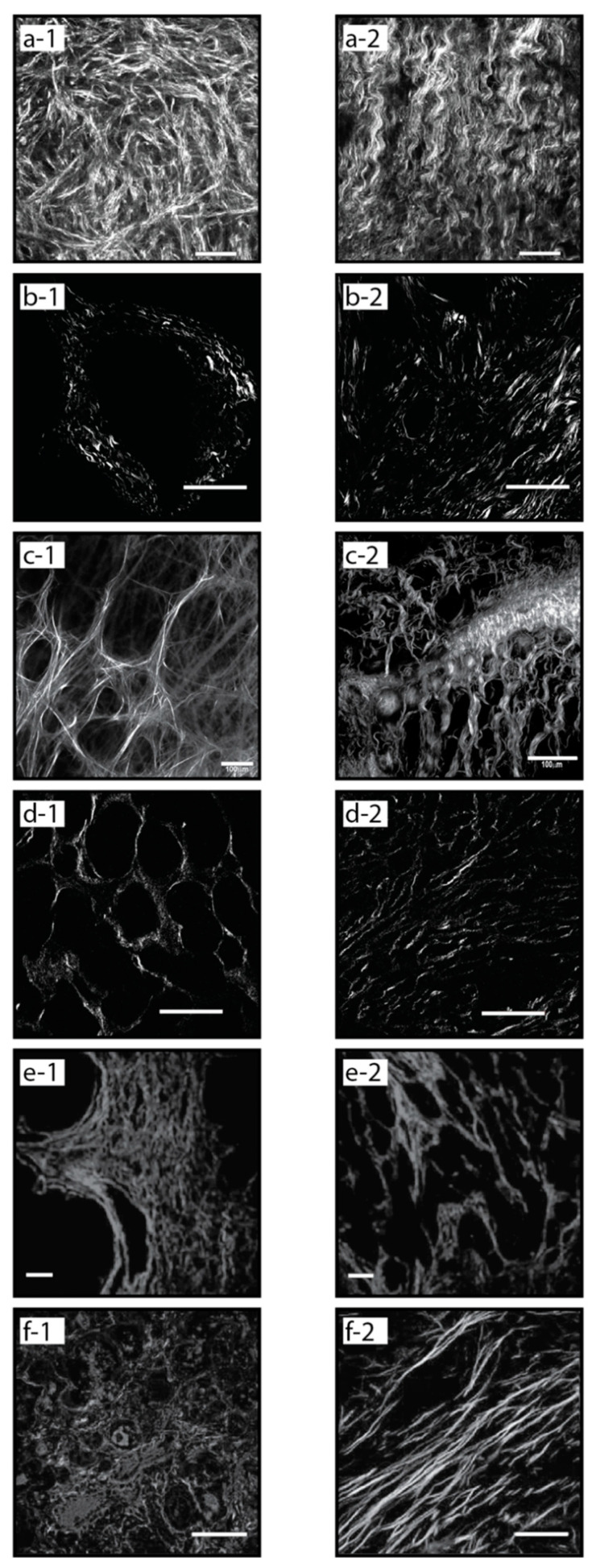
Normal vs. malignant collagen organization, Second Harmonic Generation (SHG) images from different cancer types showing characteristic signatures: normal (**a-1**) and malignant (**a-2**) ovarian tissue [9], normal (**b-1**) and high grade (**b-2**) pancreatic (PDAC) tissue [59], normal (**c-1**) and malignant (**c-2**) breast tissue, low grade (**d-1**) and high grade (**d-2**) kidney (RCC) tissue [94], normal (**e-1**) and high grade (**e-2**) prostate tissue [93], normal (**f-1**) and malignant (**f-2**) esophageal tissue [10].

Many of the methods used for quantifying morphological features of collagen are highly sensitive to detect features that cannot be elucidated by human visual analysis. In some cases, pathologists are faced with differentiating benign from malignant processes—such as chronic pancreatitis from invasive adenocarcinoma—on the basis of scant tissue with rare or distorted epithelial elements [45]. By traditional assessment methods, the stroma alone is not helpful in making this distinction. Visual or computational methods that can clearly differentiate benign from malignant collagen fiber orientation may be beneficial in these cases. The characteristic differences in collagen organization between normal and malignant tissues are shown in Figure 3 for multiple cancer types.

Collagen fiber organization in ovarian cancer is a growing area of study. Multiple groups have found that collagen fibers are more organized in high-grade serous compared to normal tissue, and evidence that significant differences in the distribution and organization of collagen fibers between various grades of ovarian tumors compared to benign and normal ovary tissue [106,108,109]. Characteristic examples of these differences in collagen fiber organization in normal and cancerous tissue are shown in Figure 3a-1,a-2, respectively.

A prospective grading application for fibrillar collagen quantification is in renal cell carcinoma (RCC). SHG-based quantification of fiber topology in RCC human samples has shown the ability to differentiate normal from cancer tissues [112], and perhaps more significantly, low-grade from high-grade tumors (Figure 3d-1,d-2) [94]. Increased fiber alignment associated with higher grading in prostate cancer (Figure 3e-1,e-2) [93]. Similarly, quantification of fibrillar collagen alignment to differentiate low-grade from high-grade cancers has been demonstrated in colorectal cancer [96].

### 3.2. Patient Prognosis

Assessment of the aggressiveness of a tumor (grade) and the extent to which it has spread through the body (stage) is the basis for prognosis and treatment recommendations in every new cancer diagnosis. Accurate pathologic analysis of biopsy or resection tissue is therefore crucial for planning patient management. A common weakness in the current systems used by pathologists to grade and stage cancers is inter-observer variability, for example, in assigning Gleason scores in prostatic cancer [113]. Quantification of collagen fiber alignment in prostatic cancer with SHG microscopy can more accurately define Gleason scores, notably, intermediate Gleason scores that are the most challenging for a pathologist to classify [93,111].

While cancer stage is supposed to correlate with prognosis, this is not always the case and additional methods for improving cancer staging are constantly evolving. As illustrated in Table 1, the potential for collagen fiber topology to provide additional prognostic information independent of traditional assessments used for grading and staging in a variety of cancer types has been amply demonstrated. Studies on collagen topology features have revealed candidate biomarkers that could be used to refine prognostic classifications. 

Breast cancer research was an early pioneer of fibrillar collagen-based prognostic studies, with the discovery of Tumor-Associated Collagen Signatures (TACS). In particular, TACS type 3 (TACS-3) in which multiple collagen fibers are bundled and aligned perpendicular to the tumor boundary, has been shown to be prognostically significant in breast cancer. Specifically, such aligned collagen fibers are a negative prognostic factor. TACS-3 corresponds to sites of focal invasion into the stroma, suggesting that tumor cells preferentially invade along these straightened, aligned collagen fibers [8,46]. The characteristic differences in collagen fiber organization between normal and cancerous breast tissue are shown in Figure 3c-1,c-2, respectively. Increased collagen fiber alignment has also been shown to be a negative prognostic factor in pancreatic ductal adenocarcinoma (PDAC) (Figure 3b-1,b-2) [59].

Collagen fiber length and width have also been investigated as prognostic factors. Increased collagen fiber length was shown to correlate with poor patient survival in multiple tumor types including head & neck squamous cell carcinoma (HNSCC), esophageal adenocarcinoma (EAC) (Figure 3f-1,f-2), and colorectal adenocarcinoma (CRC) [10]. Collagen organization in gastric cancer has been studied with collagen width as the strongest predictor of 5-year overall patient survival [88]. Using Picrosirius red to study collagen architecture and reorganization in gastric cancer patients, they were able to classify collagen organization into previously indistinguishable subgroups, with wide collagen fiber groups having higher rates of recurrence.

Most of the research on fibrillar collagen organization in cancer has focused on epithelial tumors. However, there has been growing interest in the role of fibrillar organization in neurologic tissues. While fibrillar collagen in the brain is thought to be primarily associated with blood vessels, there is growing evidence of a strong fibrotic response to wounds [114]. Unlike epithelial tissues where the primary collagen is produced by fibroblasts, it is thought that the fibrillar collagen produced in response to brain wounding events is made by immune cell types such as macrophages and microglia [115]. More aligned collagen was shown to be a positive prognostic biomarker in glioblastoma (GBM). GBM patients with more organized collagen had a longer median survival than those with less organized collagen [95]. This is the opposite of what has been reported from epithelial cancers, where more aligned collagen is negatively prognostic. More research is needed to investigate the possible reasons underlying this difference, which may be linked to the respective roles of fibroblasts versus immune cells in collagen expression and possible differential roles in the tumor/brain microenvironment.

## 4. Clinical Implications

Collagen alignment and the pathways of cell migration in the context of an organized stroma are not considered prognostically meaningful in pathology practice. The research presented here, along with growing understanding of the epithelial–mesenchymal transition, altered gene expression profiles and altered cell signaling in malignant stroma, and the role of stem cells in promoting malignant growth and metastasis are challenging the assumption that the stroma is inert and not involved in tumor progression. The repeatedly demonstrated observation that collagen organization in malignant tissue correlates with patient survival requires better understanding for translation into clinically relevant applications. The inclusion of collagen topology in pathological assessment could result in improved patient outcomes by informing patient care decisions and cancer management plans. Subtle changes can be predictive of aggressive behavior and may powerfully inform and change a patient’s treatment plan. 

The studies that have been performed to date on collagen topology in cancer tissues are not readily translated into clinical practice. A major practical hurdle is the current need for patient biopsies. Another difficulty lies in the inability to consistently compare methodologies. Many studies have used computational algorithms on tissue analyzed by SHG to define the collagen-based changes such as the TACS signature. While the TACS signature could reproducibly be determined with picrosirius red, it is still not clear from these studies how to “see” the signature in routine clinical practice. Other studies found a correlation between “straight” vs. “aligned” collagen in cancer tissues, but the relationship of “straight” vs. “aligned” collagen to TACS needs to be better defined. The distribution and alignment of collagen in tumors is typically heterogeneous, and it remains to be demonstrated whether differences in TACS or collagen alignment in the center or edge of the tumor are prognostically significant. By definition, TACS describes the orientation of collagen fibers in relation to individual malignant glands or cells, yet other studies have shown that collagen orientation by itself, without regard to the epithelial component, carries prognostic meaning. Further studies are required to develop a lexicon to reproducibly describe changes in stromal collagen so that larger studies to compare similar features may be performed. With the methodologies described in Section 2 that enable visualization and quantification of collagen orientation on routine tissue sections, collagen signatures that are prognostically meaningful can be described and harnessed to improve prognostication in a wide range of cancers.

While oncologic pathology is the most obvious field in which discovery of meaningful collagen organization patterns could contribute to improved clinical care, there are other possible clinical applications for collagen biomarkers. These areas include surgery, radiotherapy, and drug studies. There has been little investigation of fibrillar collagen in these areas, largely due to the emerging understanding of the role of collagen fiber organization in cancer growth and development. There is a growing body of research on collagen organization (using in vitro and in vivo animal models) that explore the potential for drugs to inhibit collagen formation or disrupt existing anisotropic collagen matrices to inhibit metastasis [116]. There is growing interest in exploiting these findings to investigate specific drug target for mediating alignment of collagen matrices or even to attack the collagen backbone directly. While this is a nascent study area, some drugs are already known to target the collagen backbone and fibrillar collagen-related proteins. For example, the chemical mediator of inflammation cyclooxygenase 2 (COX-2) was shown to affect fibrillar collagen in breast cancer [99]. Treatment with the COX-2 inhibitor Celecoxib was shown to reduce the collagen-dense tumor burden in glioblastoma, and to enhance radiosensitivity of hypoxic glioblastoma cells [117]. Tenascin-C and Thrombospondin-2 co-localize with aligned collagen, suggesting that they may function to structurally support collagen alignment [118]. These matricellular proteins could serve as either targets for inhibition or for development of therapeutic agents [119]. Other suggested treatments of collagen-modifying agents (e.g., LOXL2 and MMP inhibitors) or stromal depleting strategies (e.g., PEGPH20 and Hh inhibition) have yet to find success in clinical trials [120,121,122,123], perhaps indicating that a more detailed examination of feedback between collagen architecture and the microenvironment is necessary in some pathologies. Alternative approaches to disrupt aberrant collagen architecture and the tumor stroma more generally focus on interventions to the collagen-producing/organizing cells themselves, myofibroblasts (e.g., all-trans retinoic acid [124]), or the cancer cell-ECM biomechanical interaction (e.g., integrin inhibitors [125]). Addressing the fibrotic environment and targeting of collagen may also be key to disrupting immunosuppression and boosting the efficacy of immunotherapies [126]. Particular changes or characteristics of collagen topology might be useful for surgical guidance, targeting radiation, or playing a role in chemotherapy or immunotherapy strategies. 

Despite the growing body of evidence describing the importance of fibrillar collagen in cancer invasion and progression, the mechanisms of collagen deposition and alignment in the ECM are not yet fully understood. There is an abundance of published evidence related to a wide range of cancers, demonstrating that collagen organization is an important feature of disease etiology and progression and in some cases may even be a candidate biomarker (Table 1). We have focused specifically on the fiber topology itself. However, to fully realize the potential of these findings, it is important to perform studies investigating the mechanisms that govern collagen organization and structure. In breast cancer, for example, it is known that collagen alignment contributes to intravasation and the escape of metastatic cells into the blood and lymphatic systems [8,40,46], but the precise mechanisms that control this collagen fiber alignment are still largely unknown. 

Cell-to-cell interactions also play an important role in the development and spread of cancer. The spatial distribution of cancer cells in relation to stromal cells, immune cells, and molecules of the ECM have been widely studied as predictors of tumor progression and metastasis [127]. While some of these interactions may alter the collagen architecture, collagen itself plays a key role in directing and changing the ECM in ways that can both promote and inhibit tumor progression [128]. Understanding the mechanisms underlying the alignment of collagen fibers in the ECM has implications for the development of pharmacological targets that may inhibit invasion and metastasis of cancer. Recent research suggests there are possible interactions between inflammatory and immune cells, particularly macrophages, with fibroblasts in the process of alignment in the ECM [129].

More research is also needed on the distribution of the collagen topology in tissue biopsy specimens. Much of the current published work on pattern of collagen orientation has been adjacent to the tumor. However more recent data shows collagen organization changes distal to the tumor site as well [89]. Further studies are needed to examine this issue and may open new avenues of investigation for the hypothesis that collagen fibers are involved in creating a possible metastatic highway. The role of collagen topology in cancer is still a relatively new area of study, but if the prognostic significance of cancer-associated collagen characteristics continues to hold true then important clinical applications should be developed.

Collectively, these studies examined in this review demonstrate that collagen topology plays an important role in tumor progression and, if we could learn to “read” it in clinical practice, could become a powerful biomarker that would allow more precise prognostication of individual patient tumors in clinical practice. More translational work is needed to validate collagen morphometrics as useful, practical clinical biomarkers. In addition to the technical hurdles discussed, it will take additional validation studies and education to achieve widespread clinical acceptance and adoption.

## 5. Conclusions

Fibrillar collagen is known to be a major structural feature of the ECM and is essential for normal tissue development and integrity. The role of fibrillar collagen in disease, specifically in oncologic diseases, is less well understood even though the ECM often forms a major component of the tumor microenvironment. There is growing evidence that changes in fibrillar collagen organization greatly contribute to the important role of the ECM in cancer invasion and progression. The convergence of modern imaging methods and advanced computational methods has facilitated early forays into the investigation and quantitation of the role of fibrillar collagen in cancer. To date, the role of collagen fiber organization has been studied in approximately a dozen different cancers. All of these studies have shown some correlation between fiber features such as angle and alignment with disease and progression or patient outcomes. Many of these changes could help to more precisely or quantitatively define tumor stage as it correlates with survival. While the precise pathophysiologic mechanisms underlying these changes in collagen characteristics still need to be understood, the clinical utility of these observations is already under research investigation. There is a great opportunity for understanding the clinical meaning of these collagen changes in pathology, surgery, pharmacology, radiation therapy, and radiology. There is a great benefit to investigate fibrillar collagen organization as a candidate biomarker for a given cancer and also to compare fibrillar collagen characteristics across a broad spectrum of cancers. Such characterization of similarities and differences in collagen organization, expression, and visualization between cancer types may reveal new research opportunities and potential new clinical targets. 

## Figures and Tables

**Table 1:** Overview of Published Collagen Fiber Organization Studies in Cancer.

Disease	Metric	Visualization	Findings	References
Breast cancer (p)	Intensity, area, density, collagen reticular index (junctions:length), fiber length, fiber thickness	SHG	Increased amount of aggregated collagen in the tissue, brighter collagen fibers, are associated with increased survival.	[92]
Breast cancer (p)	COX2, TAMs, collagen alignment	SHG, Masson’s Trichrome, Immunofluorescence	Collagen orientation perpendicular to the tumor boundary is associated with poor overall survival.	[86,99]
Breast cancer (d)	Fiber shape	SHG	Shape different between normal, benign, and malignant breast.	[100]
Breast cancer (d)	Relative angle	SHG	TACS as a function of disease progression: More aligned collagen associated with invasion.	[8]
Breast cancer (d)	d_15_/d_31_ tensor element ratio of the second order susceptibility χ^2^	SHG	Malignant tissue contains locally aligned fibers compared to normal, hyperplastic, and dysplastic tissues.	[101]
Breast cancer (p)	Relative angle	SHG	Radially aligned fibrillar collagen is a poor prognostic factor.	[46]
Breast cancer (xenograft)	Alignment	SHG	Significantly less aligned collagen after trastuzumab treatment.	[53]
Colon Cancer (d)	Fiber width, straightness, alignment	SHG, EM	Changes in collagen alignment are apparent 10-20cm from the tumor. Increase in collagen width, straightness, and alignment further from the tumor.	[89]
Colorectal cancer (d)	Average intensity per pixel (AIPP) and fiber alignment	SHG	SHG signal intensity can differentiate malignant from non-malignant colonic polyp tissue with high sensitivity and specificity. Anisotropic polarization can discern high-grade dysplasia from normal colonic polyp tissue.	[96,102]
Esophageal Cancer (p)	Length	SHG	Elongated collagen fibers are associated with poorer prognosis, alignment did not predict survival.	[10]
Gastric cancer (d)	Average intensity per pixel	MPM, SHG	Increase in overall collagen intensity in early gastric cancer vs normal	[103]
Gastric cancer (d)	CT-FIRE (alignment, length, straightness, width),	SHG, IHC (ColI, PICP, PINP, LOX, LOXL2)	Increased stromal collagen alignment, length, straightness, and width in gastric cancer.	[88]
Glioblastoma (p)	Alignment	SHG	More organized collagen is associated with better prognosis.	[95]
Head and neck, (p)	Length	SHG	Elongated collagen fibers are associated with poorer prognosis, alignment did not predict survival.	[10]
Hepatocellular carcinoma (d)	Aggregated and distributed collagen fiber ratio, individual percentage, number, length, width, and cross-link density	SHG	Collagen architecture varies with different grades of HCC; can be used to accurately predict HCC grading.	[90]
Lung cancer	Alignment	SHG	Aligned collagen prevents T cells from migrating to tumor islets.	[13]
Non-small cell lung carcinoma (d)	second-order susceptibility component ratio	Polarization-in, polarization-out SHG	Collagen in less compact and has larger disorder in tumor tissue.	[104]
Oral squamous cell carcinoma (d)	Fiber thickness	Picrosirius Red	Thin fibers increased and thick fibers decreased with increasing grade.	[91]
Oral squamous cell carcinoma (d)	Alignment	Picrosirius Red	Parallel fibers in neoplastic regions.	[105]
Osteosarcoma, breast cancer, melanoma (d)	d22 coefficient and anisotropy	SHG	Structure different in tumor tissue, low collagen density correlates with cancer.	[106]
Ovarian cancer (d)	Gray-level co-occurrence matrix to measure fibril size and separation	SHG, TPEF	Normal tissue is more highly structured. Loss of fine structure and structural organization with wavy collagen fibers in ovarian cancer.	[107]
Ovarian cancer (d)	SHG emission attributes (directionality and relative intensity) and bulk optical parameters	SHG	Malignant tissue has higher fiber regularity.	[9]
Ovarian cancer (d)	Alignment of collagen fibers, anisotropy, and correlation	SHG	Significant differences in the distribution and organization of collagen fibers in the stroma component of serous, mucinous, endometrioid, and mixed ovarian tumors as compared with normal ovary tissue.	[108]
Ovarian cancer (d)	Intensity, scattering, anisotropy	SHG, Optical Scattering	Collagen fibers are more organized in high-grade serous compared to normal tissue.	[109]
Pancreatic ductal adenocarcinoma (p)	Fiber alignment	SLIM	Inverse relationship between survival data and fiber width and length.	[97]
Pancreatic ductal adenocarcinoma (p)	Alignment	SHG	High alignment is a poor prognostic factor.	[59,110]
Pancreatic ductal adenocarcinoma (d)	Alignment	SHG	Increased alignment, length, and width in PDAC vs. normal and chronic pancreatitis.	[45]
Prostate cancer (d)	Fiber orientation, anisotropy	SHG, US	Increased fiber alignment associated with higher Gleason score.	[111]
Prostate cancer (d)	Trained CNN using SHG	SHG, CNN	TPEF and SHG can be used in combination with deep learning for accurate and automated Gleason grading of unstained prostate tissues.	[82]
Prostate cancer (d)	Anisotropy vs Gleason (ImageJ and FibrilTool)	SHG	Increased fiber alignment associated with higher cancer grading.	[93]
Renal cell carcinoma (d)	Alignment and density	SHG	Increased fiber density and alignment in RCC grade 4 compared to grade 1.	[94]

(p)–Prognostic, (d)–Diagnostic.

## Data Availability

Not applicable.

## References

[1] Ricard-Blum S. (2011). The Collagen Family. Cold Spring Harb. Perspect. Biol..

[2] Brinckmann J. (2005). Collagens at a Glance. Top. Curr. Chem..

[3] Gautieri A., Vesentini S., Redaelli A., Buehler M.J. (2011). Hierarchical Structure and Nanomechanics of Collagen Microfibrils from the Atomistic Scale Up. Nano Lett..

[4] Bhattacharjee A., Bansal M. (2005). Collagen Structure: The Madras Triple Helix and the Current Scenario. IUBMB Life.

[5] Makareeva E., Han S., Vera J.C., Sackett D.L., Holmbeck K., Phillips C.L., Visse R., Nagase H., Leikin S. (2010). Carcinomas Contain a Matrix Metalloproteinase-Resistant Isoform of Type I Collagen Exerting Selective Support to Invasion. Cancer Res..

[6] Mouw J.K., Ou G., Weaver V.M. (2014). Extracellular Matrix Assembly: A Multiscale Deconstruction. Nat. Rev. Mol. Cell Biol..

[7] LaComb R., Nadiarnykh O., Campagnola P.J. (2008). Quantitative Second Harmonic Generation Imaging of the Diseased State Osteogenesis Imperfecta: Experiment and Simulation. Biophys. J..

[8] Provenzano P.P., Eliceiri K.W., Campbell J.M., Inman D.R., White J.G., Keely P.J. (2006). Collagen Reorganization at the Tumor-Stromal Interface Facilitates Local Invasion. BMC Med..

[9] Nadiarnykh O., LaComb R.B., Brewer M.A., Campagnola P.J. (2010). Alterations of the Extracellular Matrix in Ovarian Cancer Studied by Second Harmonic Generation Imaging Microscopy. BMC Cancer.

[10] Hanley C.J., Noble F., Ward M., Bullock M., Drifka C., Mellone M., Manousopoulou A., Johnston H.E., Hayden A., Thirdborough S. (2015). A Subset of Myofibroblastic Cancer-Associated Fibroblasts Regulate Collagen Fiber Elongation, Which Is Prognostic in Multiple Cancers. Oncotarget.

[11] Provenzano P.P., Inman D.R., Eliceiri K.W., Trier S.M., Keely P.J. (2008). Contact Guidance Mediated Three-Dimensional Cell Migration Is Regulated by Rho/ROCK-Dependent Matrix Reorganization. Biophys. J..

[12] Riching K.M., Cox B.L., Salick M.R., Pehlke C., Riching A.S., Ponik S.M., Bass B.R., Crone W.C., Jiang Y., Weaver A.M. (2014). 3D Collagen Alignment Limits Protrusions to Enhance Breast Cancer Cell Persistence. Biophys. J..

[13] Salmon H., Franciszkiewicz K., Damotte D., Dieu-Nosjean M.C., Validire P., Trautmann A., Mami-Chouaib F., Donnadieu E. (2012). Matrix Architecture Defines the Preferential Localization and Migration of T Cells into the Stroma of Human Lung Tumors. J. Clin. Investig..

[14] Shi Q., Ghosh R.P., Engelke H., Rycroft C.H., Cassereau L., Sethian J.A., Weaver V.M., Liphardt J.T. (2014). Rapid Disorganization of Mechanically Interacting Systems of Mammary Acini. Proc. Natl. Acad. Sci. USA.

[15] Wozniak M.A., Desai R., Solski P.A., Der C.J., Keely P.J. (2003). ROCK-Generated Contractility Regulates Breast Epithelial Cell Differentiation in Response to the Physical Properties of a Three-Dimensional Collagen Matrix. J. Cell Biol..

[16] Ma X., Schickel M.E., Stevenson M.D., Sarang-Sieminski A.L., Gooch K.J., Ghadiali S.N., Hart R.T. (2013). Fibers in the Extracellular Matrix Enable Long-Range Stress Transmission between Cells. Biophys. J..

[17] Levental K.R., Yu H., Kass L., Lakins J.N., Egeblad M., Erler J.T., Fong S.F.T., Csiszar K., Giaccia A., Weninger W. (2009). Matrix Crosslinking Forces Tumor Progression by Enhancing Integrin Signaling. Cell.

[18] Gehler S., Baldassarre M., Lad Y. (2009). Filamin A–Β1 Integrin Complex Tunes Epithelial Cell Response to Matrix Tension. Mol. Biol. Cell.

[19] Navab R., Strumpf D., To C., Pasko E., Kim K.S., Park C.J., Hai J., Liu J., Jonkman J., Barczyk M. (2015). Integrin A11β1 Regulates Cancer Stromal Stiffness and Promotes Tumorigenicity and Metastasis in Non-Small Cell Lung Cancer. Oncogene.

[20] Plodinec M., Loparic M., Monnier C.A., Obermann E.C., Zanetti-Dallenbach R., Oertle P., Hyotyla J.T., Aebi U., Bentires-Alj M., Lim R.Y.H. (2012). The Nanomechanical Signature of Breast Cancer. Nat. Nanotechnol..

[21] Tung J.C., Barnes J.M., Desai S.R., Sistrunk C., Conklin M.W., Schedin P., Eliceiri K.W., Keely P.J., Seewaldt V.L., Weaver V.M. (2015). Tumor Mechanics and Metabolic Dysfunction. Free Radic. Biol. Med..

[22] McConnell J.C., O’Connell O.V., Brennan K., Weiping L., Howe M., Joseph L., Knight D., O’Cualain R., Lim Y., Leek A. (2016). Increased Peri-Ductal Collagen Micro-Organization May Contribute to Raised Mammographic Density. Breast Cancer Res. BCR.

[23] Stylianopoulos T., Martin J.D., Chauhan V.P., Jain S.R., Diop-Frimpong B., Bardeesy N., Smith B.L., Ferrone C.R., Hornicek F.J., Boucher Y. (2012). Causes, Consequences, and Remedies for Growth-Induced Solid Stress in Murine and Human Tumors. Proc. Natl. Acad. Sci. USA.

[24] Jacobetz M.A., Chan D.S., Neesse A., Bapiro T.E., Cook N., Frese K.K., Feig C., Nakagawa T., Caldwell M.E., Zecchini H.I. (2012). Hyaluronan Impairs Vascular Function and Drug Delivery in a Mouse Model of Pancreatic Cancer. Gut.

[25] Gaggioli C., Hooper S., Hidalgo-Carcedo C., Grosse R., Marshall J.F., Harrington K., Sahai E. (2007). Fibroblast-Led Collective Invasion of Carcinoma Cells with Differing Roles for RhoGTPases in Leading and Following Cells. Nat. Cell Biol..

[26] Yang N., Mosher R., Seo S., Beebe D., Friedl A. (2011). Syndecan-1 in Breast Cancer Stroma Fibroblasts Regulates Extracellular Matrix Fiber Organization and Carcinoma Cell Motility. Am. J. Pathol..

[27] Morris R.A., Damon B., Mironov V., Kasyanov V., Ramamurthi A., Moreno-Rodriguez R., Trusk T., Potts J.D., Goodwin R.L., Davis J. (2007). Periostin Regulates Collagen Fibrillogenesis and the Biomechanical Properties of Connective Tissues. J. Cell. Biochem..

[28] Chen J., Chen Z., Chen M., Li D., Li Z., Xiong Y., Dong J., Li X. (2009). Role of Fibrillar Tenascin-C in Metastatic Pancreatic Cancer. Int. J. Oncol..

[29] Velling T., Risteli J., Wennerberg K., Mosher D.F., Johansson S. (2002). Polymerization of Type I and III Collagens Is Dependent on Fibronectin and Enhanced by Integrins alpha 11beta 1 and alpha 2beta 1. J. Biol. Chem..

[30] McDonald J.A., Kelley D.G., Broekelmann T.J. (1982). Role of Fibronectin in Collagen Deposition: Fab’ to the Gelatin-Binding Domain of Fibronectin Inhibits Both Fibronectin and Collagen Organization in Fibroblast Extracellular Matrix. J. Cell Biol..

[31] Sottile J., Shi F., Rublyevska I., Chiang H.-Y., Lust J., Chandler J. (2007). Fibronectin-Dependent Collagen I Deposition Modulates the Cell Response to Fibronectin. Am. J. Physiol. Cell Physiol..

[32] Kadler K.E., Hill A., Canty-Laird E.G. (2008). Collagen Fibrillogenesis: Fibronectin, Integrins, and Minor Collagens as Organizers and Nucleators. Curr. Opin. Cell Biol..

[33] Junqueira L.C.U., Bignolas G., Brentani R.R. (1979). Picrosirius Staining plus Polarization Microscopy, a Specific Method for Collagen Detection in Tissue Sections. Histochem. J..

[34] Drifka C.R., Loeffler A.G., Mathewson K., Mehta G., Keikhosravi A., Liu Y., Lemancik S., Ricke W.A., Weber S.M., Kao W.J. (2016). Comparison of Picrosirius Red Staining with Second Harmonic Generation Imaging for the Quantification of Clinically Relevant Collagen Fiber Features in Histopathology Samples. J. Histochem. Cytochem..

[35] Lattouf R., Younes R., Lutomski D., Naaman N., Godeau G., Senni K., Changotade S. (2014). Picrosirius Red Staining: A Useful Tool to Appraise Collagen Networks in Normal and Pathological Tissues. J. Histochem. Cytochem..

[36] Hwang J., Huang Y., Burwell T.J., Peterson N.C., Connor J., Weiss S.J., Yu S.M., Li Y. (2017). In Situ Imaging of Tissue Remodeling with Collagen Hybridizing Peptides. ACS Nano.

[37] Li Y., Yu S.M., Sagi I., Afratis N.A. (2019). In Situ Detection of Degraded and Denatured Collagen via Triple Helical Hybridization: New Tool in Histopathology. Collagen: Methods and Protocols.

[38] Brisson B.K., Mauldin E.A., Lei W., Vogel L.K., Power A.M., Lo A., Dopkin D., Khanna C., Wells R.G., Puré E. (2015). Type III Collagen Directs Stromal Organization and Limits Metastasis in a Murine Model of Breast Cancer. Am. J. Pathol..

[39] Keikhosravi A., Bredfeldt J.S., Sagar A.K., Eliceiri K.W., Waters J.C., Wittman T. (2014). Chapter 28—Second-harmonic generation imaging of cancer. Methods in Cell Biology.

[40] Provenzano P.P., Eliceiri K.W., Keely P.J. (2009). Shining New Light on 3D Cell Motility and the Metastatic Process. Trends Cell Biol..

[41] Provenzano P.P., Eliceiri K.W., Keely P.J. (2009). Multiphoton Microscopy and Fluorescence Lifetime Imaging Microscopy (FLIM) to Monitor Metastasis and the Tumor Microenvironment. Clin. Exp. Metastasis.

[42] Campagnola P.J. (2011). Second Harmonic Generation Imaging Microscopy: Applications to Diseases Diagnostics. Anal. Chem..

[43] Campagnola P.J., Loew L.M. (2003). Second-Harmonic Imaging Microscopy for Visualizing Biomolecular Arrays in Cells, Tissues and Organisms. Nat. Biotechnol..

[44] Condeelis J., Segall J.E. (2003). Intravital Imaging of Cell Movement in Tumours. Nat. Rev. Cancer.

[45] Drifka C.R., Tod J., Loeffler A.G., Liu Y., Thomas G.J., Eliceiri K.W., Kao W.J. (2015). Periductal Stromal Collagen Topology of Pancreatic Ductal Adenocarcinoma Differs from That of Normal and Chronic Pancreatitis. Mod. Pathol..

[46] Conklin M.W., Eickhoff J.C., Riching K.M., Pehlke C.A., Eliceiri K.W., Provenzano P.P., Friedl A., Keely P.J. (2011). Aligned Collagen Is a Prognostic Signature for Survival in Human Breast Carcinoma. Am. J. Pathol..

[47] Drifka C.R., Eliceiri K.W., Weber S.M., Kao W.J. (2013). A Bioengineered Heterotypic Stroma-Cancer Microenvironment Model to Study Pancreatic Ductal Adenocarcinoma. Lab. Chip.

[48] Ajeti V., Nadiarnykh O., Ponik S.M., Keely P.J., Eliceiri K.W., Campagnola P.J. (2011). Structural Changes in Mixed Col I/Col V Collagen Gels Probed by SHG Microscopy: Implications for Probing Stromal Alterations in Human Breast Cancer. Biomed. Opt. Express.

[49] Sung K.E., Su G., Pehlke C., Trier S.M., Eliceiri K.W., Keely P.J., Friedl A., Beebe D.J. (2009). Control of 3-Dimensional Collagen Matrix Polymerization for Reproducible Human Mammary Fibroblast Cell Culture in Microfluidic Devices. Biomaterials.

[50] Sung K.E., Yang N., Pehlke C., Keely P.J., Eliceiri K.W., Friedl A., Beebe D.J. (2011). Transition to Invasion in Breast Cancer: A Microfluidic in Vitro Model Enables Examination of Spatial and Temporal Effects. Integr. Biol..

[51] Raub C.B., Suresh V., Krasieva T., Lyubovitsky J., Mih J.D., Putnam A.J., Tromberg B.J., George S.C. (2007). Noninvasive Assessment of Collagen Gel Microstructure and Mechanics Using Multiphoton Microscopy. Biophys. J..

[52] Brown E., McKee T., di Tomaso E., Pluen A., Seed B., Boucher Y., Jain R.K. (2003). Dynamic Imaging of Collagen and Its Modulation in Tumors in Vivo Using Second-Harmonic Generation. Nat. Med..

[53] Walsh A.J., Cook R.S., Lee J.H., Arteaga C.L., Skala M.C. (2015). Collagen Density and Alignment in Responsive and Resistant Trastuzumab-Treated Breast Cancer Xenografts. J. Biomed. Opt..

[54] Oldenbourg R., Mei G. (1995). New Polarized Light Microscope with Precision Universal Compensator. J. Microsc..

[55] Oldenbourg R., Goldman R.D., Spector D.L. (2005). Polarization Microscopy with the LC-PolScope. Live Cell Imaging: A Laboratory Manual.

[56] Keikhosravi A., Liu Y., Drifka C., Woo K.M., Verma A., Oldenbourg R., Eliceiri K.W. (2017). Quantification of Collagen Organization in Histopathology Samples Using Liquid Crystal Based Polarization Microscopy. Biomed. Opt. Express.

[57] Provenzano P.P., Inman D.R., Eliceiri K.W., Knittel J.G., Yan L., Rueden C.T., White J.G., Keely P.J. (2008). Collagen Density Promotes Mammary Tumor Initiation and Progression. BMC Med..

[58] Shea M.P., O’Leary K.A., Wegner K.A., Vezina C.M., Schuler L.A. (2018). High Collagen Density Augments MTOR-Dependent Cancer Stem Cells in ERα+ Mammary Carcinomas, and Increases MTOR-Independent Lung Metastases. Cancer Lett..

[59] Drifka C.R., Loeffler A.G., Mathewson K., Keikhosravi A., Eickhoff J.C., Liu Y., Weber S.M., Kao W.J., Eliceiri K.W. (2016). Highly Aligned Stromal Collagen Is a Negative Prognostic Factor Following Pancreatic Ductal Adenocarcinoma Resection. Oncotarget.

[60] Chen G., Liu Y., Zhu X., Huang Z., Cai J., Chen R., Xiong S., Zeng H. (2015). Phase and Texture Characterizations of Scar Collagen Second-Harmonic Generation Images Varied with Scar Duration. Microsc. Microanal..

[61] Hu W., Li H., Fu L., Wang C., Gou S. (2012). Characterization of Collagen Fibers by Means of Texture Analysis of Second Harmonic Generation Images Using Orientation-Dependent Gray Level Co-Occurrence Matrix Method. J. Biomed. Opt..

[62] Wen B.L., Brewer M.A., Nadiarnykh O., Hocker J.D., Singh V., Mackie T.R., Campagnola P.J. (2014). Texture Analysis Applied to Second Harmonic Generation Image Data for Ovarian Cancer Classification. J. Biomed. Opt..

[63] Eekhoff J.D., Lake S.P. (2020). Three-Dimensional Computation of Fibre Orientation, Diameter and Branching in Segmented Image Stacks of Fibrous Networks. J. R. Soc. Interface.

[64] Brett E.A., Sauter M.A., Machens H.-G., Duscher D. (2020). Tumor-Associated Collagen Signatures: Pushing Tumor Boundaries. Cancer Metab..

[65] Quinn K.P., Georgakoudi I. (2013). Rapid Quantification of Pixel-Wise Fiber Orientation Data in Micrographs. J. Biomed. Opt..

[66] Rezakhaniha R., Agianniotis A., Schrauwen J.T.C., Griffa A., Sage D., Bouten C.V.C., Van De Vosse F., Unser M., Stergiopulos N. (2012). Experimental Investigation of Collagen Waviness and Orientation in the Arterial Adventitia Using Confocal Laser Scanning Microscopy. Biomech. Model. Mechanobiol..

[67] Chaudhuri S., Nguyen H., Rangayyan R.M., Walsh S., Frank C.B. (1987). A Fourier Domain Directional Filterng Method for Analysis of Collagen Alignment in Ligaments. IEEE Trans. Biomed. Eng..

[68] Kartasalo K., Pölönen R.-P., Ojala M., Rasku J., Lekkala J., Aalto-Setälä K., Kallio P. (2015). CytoSpectre: A Tool for Spectral Analysis of Oriented Structures on Cellular and Subcellular Levels. BMC Bioinform..

[69] Pourdeyhimi B., Kim H. (2002). Measuring Fiber Orientation in Nonwovens: The Hough Transform. Text. Res. J..

[70] Pourdeyhimi B., Dent R., Davis H. (1997). Measuring Fiber Orientation in Nonwovens Part III: Fourier Transform. Text. Res. J..

[71] Püspöki Z., Storath M., Sage D., Unser M., De Vos W.H., Munck S., Timmermans J.-P. (2016). Transforms and Operators for Directional Bioimage Analysis: A Survey. Focus on Bio-Image Informatics.

[72] Xu B., Yu L. (1997). Determining Fiber Orientation Distribution in Nonwovens with Hough Transform Techniques. Text. Res. J..

[73] Wu J., Rajwa B., Filmer D.L., Hoffmann C.M., Yuan B., Chiang C.-S., Sturgis J., Robinson J.P. (2003). Analysis of Orientations of Collagen Fibers by Novel Fiber-Tracking Software. Microsc. Microanal..

[74] Stein A.M., Vader D.A., Jawerth L.M., Weitz D.A., Sander L.M. (2008). An Algorithm for Extracting the Network Geometry of Three-Dimensional Collagen Gels. J. Microsc..

[75] Bredfeldt J.S., Liu Y., Pehlke C.A., Conklin M.W., Szulczewski J.M., Inman D.R., Keely P.J., Nowak R.D., Mackie T.R., Eliceiri K.W. (2014). Computational Segmentation of Collagen Fibers from Second-Harmonic Generation Images of Breast Cancer. J. Biomed. Opt..

[76] Candès E., Demanet L., Donoho D., Ying L. (2006). Fast Discrete Curvelet Transforms. Multiscale Model. Simul..

[77] Liu Y., Keikhosravi A., Pehlke C.A., Bredfeldt J.S., Dutson M., Liu H., Mehta G.S., Claus R., Patel A.J., Conklin M.W. (2020). Fibrillar Collagen Quantification With Curvelet Transform Based Computational Methods. Front. Bioeng. Biotechnol..

[78] Bredfeldt J.S. (2014). Collagen Alignment Imaging and Analysis for Breast Cancer Classification. Ph.D. Thesis.

[79] Reis L.A., Garcia A.P., Gomes E.F., Longford F.G., Frey J.G., Cassali G.D., de Paula A.M. (2020). Canine Mammary Cancer Diagnosis from Quantitative Properties of Nonlinear Optical Images. Biomed. Opt. Express.

[80] Huttunen M.J., Hassan A., McCloskey C.W., Fasih S., Upham J., Vanderhyden B.C., Boyd R.W., Murugkar S. (2018). Automated Classification of Multiphoton Microscopy Images of Ovarian Tissue Using Deep Learning. J. Biomed. Opt..

[81] Wen B., Campbell K.R., Tilbury K., Nadiarnykh O., Brewer M.A., Patankar M., Singh V., Eliceiri K.W., Campagnola P.J. (2016). 3D Texture Analysis for Classification of Second Harmonic Generation Images of Human Ovarian Cancer. Sci. Rep..

[82] Yang Q., Xu Z., Liao C., Cai J., Huang Y., Chen H., Tao X., Huang Z., Chen J., Dong J. (2020). Epithelium Segmentation and Automated Gleason Grading of Prostate Cancer via Deep Learning in Label-free Multiphoton Microscopic Images. J. Biophotonics.

[83] Lindeberg T. (1998). Edge Detection and Ridge Detection with Automatic Scale Selection. Int. J. Comput. Vis..

[84] Boudaoud A., Burian A., Borowska-Wykręt D., Uyttewaal M., Wrzalik R., Kwiatkowska D., Hamant O. (2014). FibrilTool, an ImageJ Plug-in to Quantify Fibrillar Structures in Raw Microscopy Images. Nat. Protoc..

[85] Wershof E., Barry D.J., Jenkins R.P., Rullan A., Wilkins A., Roxanis I., Anderson K.I., Park D., Bates P.A., Sahai E. (2019). A FIJI Macro for Quantifying Pattern in Extracellular Matrix. bioRxiv.

[86] Bredfeldt J.S., Liu Y., Conklin M.W., Keely P.J., Mackie T.R., Eliceiri K.W. (2014). Automated Quantification of Aligned Collagen for Human Breast Carcinoma Prognosis. J. Pathol. Inform..

[87] Liu Y., Keikhosravi A., Mehta G.S., Drifka C.R., Eliceiri K.W., Rittié L. (2017). Methods for Quantifying Fibrillar Collagen Alignment. Fibrosis: Methods and Protocols.

[88] Zhou Z.-H., Ji C.-D., Xiao H.-L., Zhao H.-B., Cui Y.-H., Bian X.-W. (2017). Reorganized Collagen in the Tumor Microenvironment of Gastric Cancer and Its Association with Prognosis. J. Cancer.

[89] Despotović S.Z., Milićević Đ.N., Krmpot A.J., Pavlović A.M., Živanović V.D., Krivokapić Z., Pavlović V.B., Lević S., Nikolić G., Rabasović M.D. (2020). Altered Organization of Collagen Fibers in the Uninvolved Human Colon Mucosa 10 Cm and 20 Cm Away from the Malignant Tumor. Sci. Rep..

[90] Lin H., Lin L., Wang G., Zuo N., Zhan Z., Xie S., Chen G., Chen J., Zhuo S. (2018). Label-Free Classification of Hepatocellular-Carcinoma Grading Using Second Harmonic Generation Microscopy. Biomed. Opt. Express.

[91] Arun Gopinathan P., Kokila G., Jyothi M., Ananjan C., Pradeep L., Humaira Nazir S. (2015). Study of Collagen Birefringence in Different Grades of Oral Squamous Cell Carcinoma Using Picrosirius Red and Polarized Light Microscopy. Scientifica.

[92] Gole L., Yeong J., Lim J.C.T., Ong K.H., Han H., Thike A.A., Poh Y.C., Yee S., Iqbal J., Hong W. (2020). Quantitative Stain-Free Imaging and Digital Profiling of Collagen Structure Reveal Diverse Survival of Triple Negative Breast Cancer Patients. Breast Cancer Res..

[93] Garcia A.M., Magalhes F.L., Soares J.S., Junior E.P., Lima M.F.R.d., Mamede M., Paula A.M.d. (2018). Second Harmonic Generation Imaging of the Collagen Architecture in Prostate Cancer Tissue. Biomed. Phys. Eng. Express.

[94] Best S.L., Liu Y., Keikhosravi A., Drifka C.R., Woo K.M., Mehta G.S., Altwegg M., Thimm T.N., Houlihan M., Bredfeldt J.S. (2019). Collagen Organization of Renal Cell Carcinoma Differs between Low and High Grade Tumors. BMC Cancer.

[95] Pointer K.B., Clark P.A., Schroeder A.B., Salamat M.S., Eliceiri K.W., Kuo J.S. (2017). Association of Collagen Architecture with Glioblastoma Patient Survival. J. Neurosurg..

[96] Birk J.W., Tadros M., Moezardalan K., Nadyarnykh O., Forouhar F., Anderson J., Campagnola P. (2014). Second Harmonic Generation Imaging Distinguishes Both High-Grade Dysplasia and Cancer from Normal Colonic Mucosa. Dig. Dis. Sci..

[97] Fanous M., Keikhosravi A., Kajdacsy-Balla A., Eliceiri K.W., Popescu G. (2020). Quantitative Phase Imaging of Stromal Prognostic Markers in Pancreatic Ductal Adenocarcinoma. Biomed. Opt. Express.

[98] Laklai H., Miroshnikova Y.A., Pickup M.W., Collisson E.A., Kim G.E., Barrett A.S., Hill R.C., Lakins J.N., Schlaepfer D.D., Mouw J.K. (2016). Genotype Tunes Pancreatic Ductal Adenocarcinoma Tissue Tension to Induce Matricellular Fibrosis and Tumor Progression. Nat. Med..

[99] Esbona K., Yi Y., Saha S., Yu M., Van Doorn R.R., Conklin M.W., Graham D.S., Wisinski K.B., Ponik S.M., Eliceiri K.W. (2018). The Presence of Cyclooxygenase 2, Tumor-Associated Macrophages, and Collagen Alignment as Prognostic Markers for Invasive Breast Carcinoma Patients. Am. J. Pathol..

[100] Falzon G., Pearson S., Murison R. (2008). Analysis of Collagen Fibre Shape Changes in Breast Cancer. Phys. Med. Biol..

[101] Ambekar R., Lau T.-Y., Walsh M., Bhargava R., Toussaint K.C.J. (2012). Quantifying Collagen Structure in Breast Biopsies Using Second-Harmonic Generation Imaging. Biomed. Opt. Express.

[102] Lukina M.M., Dudenkova V.V., Shimolina L.E., Snopova L.B., Zagaynova E.V., Shirmanova M.V. (2019). In Vivo Metabolic and SHG Imaging for Monitoring of Tumor Response to Chemotherapy. Cytom. Part A.

[103] Li L., Kang D., Huang Z., Zhan Z., Feng C., Zhou Y., Tu H., Zhuo S., Chen J. (2019). Multimodal Multiphoton Imaging for Label-Free Monitoring of Early Gastric Cancer. BMC Cancer.

[104] Golaraei A., Cisek R., Krouglov S., Navab R., Niu C., Sakashita S., Yasufuku K., Tsao M.-S., Wilson B.C., Barzda V. (2014). Characterization of Collagen in Non-Small Cell Lung Carcinoma with Second Harmonic Polarization Microscopy. Biomed. Opt. Express.

[105] Manjunatha B.S., Agrawal A., Shah V. (2015). Histopathological Evaluation of Collagen Fibers Using Picrosirius Red Stain and Polarizing Microscopy in Oral Squamous Cell Carcinoma. J. Cancer Res. Ther..

[106] Hompland T., Erikson A., Lindgren M., Lindmo T., de Lange Davies C. (2008). Second-Harmonic Generation in Collagen as a Potential Cancer Diagnostic Parameter. J. Biomed. Opt..

[107] Kirkpatrick N.D., Brewer M.A., Utzinger U. (2007). Endogenous Optical Biomarkers of Ovarian Cancer Evaluated with Multiphoton Microscopy. Cancer Epidemiol. Biomark. Prev..

[108] Adur J., Pelegati V.B., de Thomaz A.A., Baratti M.O., Andrade L.A.L.A., Carvalho H.F., Bottcher-Luiz F., Cesar C.L. (2014). Second Harmonic Generation Microscopy as a Powerful Diagnostic Imaging Modality for Human Ovarian Cancer. J. Biophotonics.

[109] Tilbury K.B., Campbell K.R., Eliceiri K.W., Salih S.M., Patankar M., Campagnola P.J. (2017). Stromal Alterations in Ovarian Cancers via Wavelength Dependent Second Harmonic Generation Microscopy and Optical Scattering. BMC Cancer.

[110] Vennin C., Chin V.T., Warren S.C., Lucas M.C., Herrmann D., Magenau A., Melenec P., Walters S.N., Monte-Nieto G.d., Conway J.R.W. (2017). Transient Tissue Priming via ROCK Inhibition Uncouples Pancreatic Cancer Progression, Sensitivity to Chemotherapy, and Metastasis. Sci. Transl. Med..

[111] Ling Y., Li C., Feng K., Palmer S., Appleton P.L., Lang S., McGloin D., Huang Z., Nabi G. (2017). Second Harmonic Generation (SHG) Imaging of Cancer Heterogeneity in Ultrasound Guided Biopsies of Prostate in Men Suspected with Prostate Cancer. J. Biophotonics.

[112] Galli R., Sablinskas V., Dasevicius D., Laurinavicius A., Jankevicius F., Koch E., Steiner G. (2014). Non-Linear Optical Microscopy of Kidney Tumours. J. Biophotonics.

[113] Burchardt M., Engers R., Müller M., Burchardt T., Willers R., Epstein J.I., Ackermann R., Gabbert H., De La Taille A., Rubin M. (2008). Interobserver Reproducibility of Gleason Grading: Evaluation Using Prostate Cancer Tissue Microarrays. J. Cancer Res. Clin. Oncol..

[114] Ogawa K., Oguchi M., Nakashima Y., Yamabe H. (1989). Distribution of Collagen Type IV in Brain Tumors: An Immunohistochemical Study. J. Neurooncol..

[115] Esquibel C.R., Wendt K.D., Lee H.C., Gaire J., Shoffstall A., Urdaneta M.E., Chacko J.V., Brodnick S.K., Otto K.J., Capadona J.R. (2020). Second Harmonic Generation Imaging of Collagen in Chronically Implantable Electrodes in Brain Tissue. Front. Neurosci..

[116] Han W., Chen S., Yuan W., Fan Q., Tian J., Wang X., Chen L., Zhang X., Wei W., Liu R. (2016). Oriented Collagen Fibers Direct Tumor Cell Intravasation. Proc. Natl. Acad. Sci. USA.

[117] Suzuki K., Gerelchuluun A., Hong Z., Sun L., Zenkoh J., Moritake T., Tsuboi K. (2013). Celecoxib Enhances Radiosensitivity of Hypoxic Glioblastoma Cells through Endoplasmic Reticulum Stress. Neuro-Oncology.

[118] Tomko L.A., Hill R.C., Barrett A., Szulczewski J.M., Conklin M.W., Eliceiri K.W., Keely P.J., Hansen K.C., Ponik S.M. (2018). Targeted Matrisome Analysis Identifies Thrombospondin-2 and Tenascin-C in Aligned Collagen Stroma from Invasive Breast Carcinoma. Sci. Rep..

[119] Sawyer A.J., Kyriakides T.R. (2016). Matricellular Proteins in Drug Delivery: Therapeutic Targets, Active Agents, and Therapeutic Localization. Adv. Drug Deliv. Rev..

[120] Jiang H., Torphy R.J., Steiger K., Hongo H., Ritchie A.J., Kriegsmann M., Horst D., Umetsu S.E., Joseph N.M., McGregor K. (2020). Pancreatic Ductal Adenocarcinoma Progression Is Restrained by Stromal Matrix. J. Clin. Investig..

[121] Lee J.J., Perera R.M., Wang H., Wu D.-C., Liu X.S., Han S., Fitamant J., Jones P.D., Ghanta K.S., Kawano S. (2014). Stromal Response to Hedgehog Signaling Restrains Pancreatic Cancer Progression. Proc. Natl. Acad. Sci. USA.

[122] Moore M.J., Hamm J., Dancey J., Eisenberg P., Dagenais M., Fields A., Hagan K., Greenberg B., Colwell B., Zee B. (2003). Comparison of Gemcitabine versus the Matrix Metalloproteinase Inhibitor BAY 12-9566 in Patients with Advanced or Metastatic Adenocarcinoma of the Pancreas: A Phase III Trial of the National Cancer Institute of Canada Clinical Trials Group. J. Clin. Oncol..

[123] Nguyen E.V., Pereira B.A., Lawrence M.G., Ma X., Rebello R.J., Chan H., Niranjan B., Wu Y., Ellem S., Guan X. (2019). Proteomic Profiling of Human Prostate Cancer-Associated Fibroblasts (CAF) Reveals LOXL2-Dependent Regulation of the Tumor Microenvironment. Mol. Cell. Proteomics.

[124] Kocher H.M., Basu B., Froeling F.E., Sarker D., Slater S., Carlin D., de Souza N.M., De Paepe K.N., Goulart M.R., Hughes C. (2020). Phase I Clinical Trial Repurposing All-Trans Retinoic Acid as a Stromal Targeting Agent for Pancreatic Cancer. Nat. Commun..

[125] Sheridan C. (2019). Pancreatic Cancer Provides Testbed for First Mechanotherapeutics. Nat. Biotechnol..

[126] Jiang H., Hegde S., Knolhoff B.L., Zhu Y., Herndon J.M., Meyer M.A., Nywening T.M., Hawkins W.G., Shapiro I.M., Weaver D.T. (2016). Targeting Focal Adhesion Kinase Renders Pancreatic Cancers Responsive to Checkpoint Immunotherapy. Nat. Med..

[127] Lu P., Weaver V.M., Werb Z. (2012). The Extracellular Matrix: A Dynamic Niche in Cancer Progression. J. Cell Biol..

[128] Xu S., Xu H., Wang W., Li S., Li H., Li T., Zhang W., Yu X., Liu L. (2019). The Role of Collagen in Cancer: From Bench to Bedside. J. Transl. Med..

[129] Park D., Wershof E., Boeing S., Labernadie A., Jenkins R.P., George S., Trepat X., Bates P.A., Sahai E. (2020). Extracellular Matrix Anisotropy Is Determined by TFAP2C-Dependent Regulation of Cell Collisions. Nat. Mater..

